# Supplementary material for: Determinants of agricultural employment during the COVID-19 pandemic: A spatial analysis of Brazilian municipalities
Source: PLoS One. 2025 Jan 9;20(1):e0316489. doi: 10.1371/journal.pone.0316489 (PMC11717285; doi:10.1371/journal.pone.0316489)
Supplement: S1 Table — ***p<0.001; **p<0.01; *p<0.05; .p<0.1. Source: Research data. (DOCX) [file pone.0316489.s001.docx]

**S1 Table 1. Model estimates in different spatial patterns. ^***^p<0.001; ^**^p<0.01; ^*^p<0.05;.p<0.1.**

| **Variables** | **SAR**  **(FE)** | **SEM**  **(FE)** | **SDM**  **(FE)** |
| --- | --- | --- | --- |
| $\rho$ |  | 0.3676^***^ |  |
|  |  | (0.0091) |  |
| $\lambda$ | 0.3586^***^ |  | 0.3488^***^ |
|  | (0.0091) |  | (0.0092) |
| $\phi$ |  |  |  |
|  |  |  |  |
| $x_{1}$ | 6.0377e-07^***^ | 7.3769e-07^***^ | 7.3427e-07^***^ |
|  | (5.3102e-08) | (5.3753e-08) | (5.3732e-08) |
| $x_{2}$ | 5.9669e-07^***^ | 9.5831e-07^***^ | 9.2043e-07^**^ |
|  | (2.8184e-07) | (2.8140e-07) | (2.8215e-07) |
| $x_{3}$ | 3.1091e-04 | 1.2964e-03^**^ | 2.6481e-03^***^ |
|  | (4.3489e-04) | (4.9692e-04) | (5.3837e-04) |
| $x_{4}$ | -1.7881e-05 | -2.5250e-05 | -1.2169e-05 |
|  | (2.6798e-05) | (2.6823e-05) | (2.6840e-05) |
| $x_{5}$ | 1.0333e-02^***^ | 8.3933e-03^***^ | 8.9709e-03^***^ |
|  | (2.3118e-03) | (2.2727e-03) | (2.3275e-03) |
| X6 | 1.5369e-04^***^ | 1.5342e-04^***^ | 1.5852e-04^***^ |
|  | (2.1939e-05) | (2.1883e-05) | (2.2109e-05) |
| X7 | -2.7508e-03^***^ | -2.8835e-03^***^ | -3.0466e-03^***^ |
|  | (5.3662e-04) | (5.4104e-04) | (5.4077e-04) |
| X8 | -5.7459e-01^***^ | -6.4666e-01^*^ | -3.8093e-01 |
|  | (2.9151e-01) | (3.2796e-01) | (3.4936e-01) |
| X9 | 6.4700e-02^***^ | 7.9172e-02^***^ | 3.3205e-02^*^ |
|  | (1.1356e-02) | (1.2543e-02) | (1.5347e-02) |
| $Wx_{1}$ |  |  | -1.1854e-06^***^ |
|  |  |  | (9.9841e-08) |
| ${Wx}_{2}$ |  |  | -3.6511e-06^***^ |
|  |  |  | (5.3986e-07) |
| ${Wx}_{3}$ |  |  | -4.5652e-03^***^ |
|  |  |  | (7.6820e-04) |
| ${Wx}_{4}$ |  |  | 1.4493e-04^**^ |
|  |  |  | (5.5986e-05) |
| $Wx_{5}$ |  |  | 9.7058e-04 |
|  |  |  | (2.3683e-03) |
| Wx6 |  |  | -3.4568e-05 |
|  |  |  | (3.9655e-05) |
| Wx7 |  |  | 9.8497e-04 |
|  |  |  | (9.3914e-04) |
| Wx8 |  |  | 6.0545e-01 |
|  |  |  | (5.0980e-01) |
| Wx9 |  |  | 1.2013e-01^***^ |
|  |  |  | (2.7023e-02) |
|  |  |  |  |
| Log-Likehood | -82,953.72 | -162,840.7 | -82,818.81 |
| $R^{2}$ | 0.8159 | 0.7983 | 0.8179 |

**Source: Research data.**

**S1 Dataset. Dataset used in the research. The variables are coded according to Table 1 of the manuscript. In addition to the variables used in the models, the panel data contains additional information representing the ID of each municipality (id), the municipality code (codmun), the name of the municipality (municipality), the state abbreviation (state_abr), and the year of observation (year).**
