# Supplementary material for: Determinants of agricultural employment during the COVID-19 pandemic: A spatial analysis of Brazilian municipalities
Source: PLoS One. 2025 Jan 9;20(1):e0316489. doi: 10.1371/journal.pone.0316489 (PMC11717285; doi:10.1371/journal.pone.0316489)
Supplement: Attachment — Submitted filename: Letter Plos One 13102024.docx [file pone.0316489.s004.docx]

Dear Dr. Carolina Serpieri and Reviewers,

We are grateful for the time and valuable contributions that each of you made to review our manuscript. Your suggestions were essential to identifying areas for improvement and increasing the robustness and clarity of our research. In response, we revised the manuscript in detail. We believe that incorporating your recommendations helped to strengthen the focus, broaden the scope of the literature review, and improve methodological accuracy, contributing to the results making a significant contribution to the topic at hand. Below is a summary of the changes made in response to the suggestions received.

Response to Editor's Comments:

Suggestion 1: “Introduction:

*The research question needs to be further clarified. For example , what is the extent of the impact of the COVID-19 pandemic on agricultural in Brazil （ Not just employment rates, because you want to highlight the value of your research ） ? Which factors have the most significant impact on agricultural employment ?*

*Consider adding an introduction to the current status of agricultural development in Brazil to better understand the research background.”*

**Answer:** We have reworded the introduction to expand the focus of the research proposal beyond the impact of the pandemic. We have also included general data that highlight the importance of the agricultural sector in the Brazilian economy, such as its contribution to the Gross Domestic Product (GDP) and formal employment. We have made the following points as suggested: we have incorporated information on the role of the agricultural sector in the Brazilian economy, we have specified more clearly the objective of the study, detailing the determinants analyzed in the variation of agricultural employment, we have justified the use of a multifactorial approach to understand the various influences on the sector. In addition, we have highlighted the importance of spatial analysis to capture regional interactions and variations in the distribution of agricultural employment.

**Suggestion 2: “ *Literature Review*** *: It is recommended to supplement some international comparative studies on the impact of the COVID-19 pandemic on agricultural employment and research on the application of spatial econometric models in agricultural employment studies. Compare the differences between their studies and the advantages of ours .*

*Consider categorizing the literature review by research themes , such as the impact of the COVID-19 pandemic on agricultural employment , factors influencing agricultural employment , and the application of spatial econometric models.”*

**Answer:** In the theoretical framework, we made a significant restructuring, organizing the literature review according to the categorization of the topics covered, as suggested. We added a specific section to discuss the results of international studies that applied spatial methods similar to ours, allowing for a comparative analysis. We also expanded the references, including more recent and relevant studies published in prominent journals. In addition, we incorporated figures and maps to enrich the content with visual elements, complementing the textual analysis.

**Suggestion 3 – “** *Research Data: The research data is comprehensive but still needs further improvement . It is recommended to explain the data collection method , the reliability of the data source , and the handling method of missing data.*

*Consider adding some methods for evaluating data quality , such as descriptive statistical analysis and data cleaning* **.”**

Response: The contributions have been incorporated. We have added two justifications for using the spatial weights matrix and completely reworked the section on spatial model selection, using Elhorst 's (2010) approach to select the model that best fits the data.

*Suggestions related to method and results. Model Selection ; Research Results : Heterogeneity Analysis : Conclusion ;*

All suggestions for methodological improvements and presentation of results have been addressed. We have explained in more detail the reliability of the data sources and the treatment given to missing data. We have also added a new section in the methodology to describe the heterogeneity and robustness tests applied, with the results presented afterwards. The conclusions have been improved to reflect these additions and strengthen the final analysis.

**Response to Reviewer 1's Comments:**

Suggestion 1: *“The study uses data from 2018 to 2021 but does not adequately justify the selection of this specific timeframe . It is unclear whether the period before 2018 might have provided a better baseline for comparison , considering the potential long-term trends in agriculture employment that were disrupted by the pandemic . This omission could lead to a misinterpretation of the pandemic's current impact .* ***”***

**Answer:** We have supplemented the specific section of the methodology “ Data description” by reinforcing the justification for choosing the time frame. We emphasize that the inclusion of periods prior to 2018 could distort the analysis of the results, given the significant effects of the crisis that hit Brazil between 2014 and 2016.
The selection of the period from 2018 to 2021 was strategically chosen with the aim of capturing both the pre-existing effects and the direct impact of the COVID-19 pandemic on the agricultural sector and on the municipal assistance policies implemented during the pandemic. The choice of this interval allows for a consistent analysis of the economic and social conditions immediately prior to the pandemic, establishing an appropriate baseline for comparison. From an analytical point of view, introducing years prior to 2018 could result in the generation of significant biases, since events such as the 2015-2016 economic crisis also had a profound impact on the agricultural sector and rural employment in Brazil, and such an effect could make it difficult to accurately distinguish the specific implications of the pandemic. Furthermore, the period covered by the work is in line with the availability of official data when the work was in the database construction phase.

**Suggestion 2:** “ *The study is centered on Brazilian municipalities , but the discussion does not sufficiently address whether or how the findings might be applicable to other contexts or countries. This limits the broader relevance of the research and its contribution to global discussions on agricultural employment during crises.* ”

**Response:** We have included a discussion of the generality of the results in the conclusion section, noting that although the focus was on Brazilian municipalities, the findings may have relevance for other developing countries with significant agricultural economies and similar social structures. Contexts in which the agricultural sector plays a key role may observe similar patterns of resilience, especially in areas where seasonal employment and exports are relevant.

However, it is important to note that the particularities of each country—such as the structure of production chains, specific public policies, and the degree of agricultural mechanization—influence how these factors interact with local employment. Thus, although the results of this research contribute to the debate on the impact of COVID-19 on the agricultural sector, their applicability to other contexts requires a careful examination of the economic and social specificities of each country.

**Suggestion 3:** “ *The paper's methodology section lacks thoroughness discussion of potential biases in the data or limitations in the spatial models used . For example , the impact of missing data or inaccuracies in reported COVID-19 cases and deaths on the results is not considered , which could affect the validity of the findings .* ”.

**Answer:** We have restructured the research variables section, highlighting the credibility of the official data sources and the technique applied to treat missing data (“ **Data description”)** . We opted for simple median imputation, given the low proportion of missing values (less than 3% per variable). This technique is less sensitive to outliers and preserves statistical power, avoiding the losses that would occur with the complete exclusion of cases, which maintains the integrity of the econometric models.

**Suggestion 4:** “ *The literature review is somewhat superficial and does not fully engage with the existing body of work on agricultural employment or the broader socio-economic impacts of pandemics ”*

**Answer:** We have completely reformulated the theoretical framework, highlighting the effects of the pandemic on agriculture, a section that addresses studies that used similar methods, the determinants of agricultural employment and a regional profile of agriculture in Brazil. In addition, we have included more current and relevant references.

**Suggestion 5:** “ *The interpretation of the results , especially regarding the impact of COVID-19 on employment , is occasionally ambiguous . The paper sometimes conflates correlation with causation , suggesting that the pandemic directly caused certain employment trends without fully substantiating these claims .* ”

**Answer:** We have comprehensively reformulated the interpretation of the results, making it clearer and unambiguous. In addition, we have further analyzed both the direct and indirect impacts for each of the variables included in the model.

**Response to Reviewer 2's Comments:**

**Suggestion 1:** “ *The paper contains several typographical and grammatical errors, poor sentence structure, and overlooks simple tenets of standard writing. Please check ..* ”

**Answer:** We carried out an extensive review of the English language, ensuring the quality of the text.

Suggestion 2: “ *Let's start with the title “The Effects of the COVID-19 Pandemic on Agricultural Employment in Brazil Municipalities” . In my opinion , the title of this manuscript is not attractive enough because it only reflects the existence of some influencing factors , but the expression of specific factors is not clear enough . In other words, the COVID-19 Pandemic is not just a simple factor . It contains a string of elements , which you have also pointed out in the following text* ”

**Answer:** We have reworded the title to: “ Determinants of Agricultural Employment During the COVID-19 Pandemic : A Spatial Analysis of Brazilian Municipalities ”. The idea is to highlight that we investigated several dimensions that influence agricultural employment, in addition to the covid-19 pandemic, as well as to show that this is an analysis that considers spatial aspects.

**Suggestion 3:** “ *The paper constantly makes use of improper pronouns such as " we " which should be avoided in scientific studies .* ”

**Answer:** We reviewed the text and removed inappropriate pronouns from the text.

**Suggestion 4:** “ *The marginal contribution of this manuscript is not clearly stated . If necessary , it is recommended to separately state the marginal contribution of the manuscript in the literature review or introduction section . Is this manuscript just applying other research methods to the data in Brazilian Municipalities ? If it's convenient , please reply to me with your thoughts .* ”.

**Answer:** In order to address the marginal contribution of the manuscript, we have made changes to the introduction to better highlight the main points. In the third paragraph, we specify that the contribution of the study is threefold. First, we investigate the socioeconomic determinants, public policies, extreme weather events, and the impact of COVID-19 on agricultural employment, something that has not been explored in the literature with a spatial approach and focused on Brazil. Second, we incorporate a spillover analysis at the municipal level, allowing us to observe the direct and indirect impacts of these factors on the spatial distribution of agricultural employment, something essential to understand local economic interactions. Finally, the analysis allows for a regional differentiation that highlights how economic characteristics and weather events heterogeneously affect agricultural employment across municipalities.

In the theoretical framework, we include a section that highlights the gap in spatial studies focused on the effects of the pandemic on agricultural employment in Brazil, a significant marginal contribution of our study.

**Suggestion 5:** “ *The references cited in this manuscript lack authoritative journal papers (eg Papers from American Economic Review ( AER), Quarterly Journal of Economics (QJE), Journal of Political Economy (JPE)). Some old references in the literature should be replaced with most recent ( past 5 years ) studies . Please check it* ”.

**Answer** : We inserted three articles from the suggested journals into the theoretical framework, namely: [Zabatantou et al. (2023)](https://www.scirp.org/journal/paperinformation?paperid=129723) , [Yeboah et al. (2021)](https://www.scirp.org/journal/paperinformation?paperid=108639) and [Fahokhi & Pellegrina (2023)](https://www.journals.uchicago.edu/doi/epdf/10.1086/724319) . In addition to these, we added numerous other references to more recent articles published in relevant journals.

**Suggestion 6:** “ *The literature review section of this manuscript lacks some content . Specifically , the authors should conduct a more in-depth analysis of the reasons for changes in labor allocation in the production sector, rather than simply summarizing the conclusions of other literature* .”

**Answer:** We have reworded the section that presents the literature review, including highlighting the issue of the migration of workers from industry and services to agriculture. To this end, we used as references the works of [Deb (2021)](https://journals.sagepub.com/doi/full/10.1177/00490857211040253) , focusing on India, and [Charlton & Castillo (2020)](https://onlinelibrary.wiley.com/doi/abs/10.1002/aepp.13105) focusing on the United States of America (USA). With regard to Brazil, we have not found any relevant studies that investigate this phenomenon to date.

**Suggestion 7** : “ *Starting from Line 215, the authors explain the calculation method of the index. But in my opinion , these are relatively basic econometric contents that are not original to the authors , and most scholars are familiar with them . In other words, they can be deleted .* ”

**Answer:** We have accepted the suggestion and simplified the section on the spatial autocorrelation index, making it more concise and straightforward, as can be seen in the section " Spatial Correlation Test: Moran's Index".

**Suggestion 8:** “ *This manuscript involves empirical research , which involves hypothesis testing . I suggest the authors clearly state before Line 200 which hypotheses are proposed in this manuscript , which will also facilitate readers ' reading . You can just use “H1: xxx ; H2: xxx ” to illustrate it* ”

**Answer:** We accept the suggestion and insert 5 research hypotheses in the sections of the theoretical framework. They are:

“H _1_ : The pandemic , in terms of the number of COVID-19 cases, positively impacted agricultural employment in Brazil . ( Impact Section of the COVID-19 Pandemic on Agricultural Employment ).

H _2_ : The effects of the COVID-19 pandemic on agricultural employment present spatial spillover effects , both at the macro and regional levels . ( Application section of Spatial Econometric Models)

H _3_ and H _4_ : Extreme weather events had a significant impact on the reduction of agricultural employment during the period and H4: The implementation of public income transfer policies during the health crisis positively influenced the maintenance of agricultural employment . ( Determinants section of Agricultural Employment ).

H _5_ : The effects of the pandemic on agricultural employment is heterogeneous , varying according to the productive characteristics of each region of the country (Regional Characteristics Section) of the Agricultural Sector in Brazil ).

In addition to the hypotheses, a figure was included (“Figure 2”) to highlight and improve visual understanding of these points.

**Suggestion 9:** “ *My question goes as: Is your suggestion universally applicable? Or just targeting the Brazilian Municipalities? Plus, the outlook for future research is not enough. It is needed to supplement and improve this section ..* ”

**Answer:** We adopted the proposed suggestions, and such considerations are set out in the conclusion of the study. Although our study focuses on Brazilian municipalities, the results may be applicable to other developing countries with similar agricultural economies, especially those in which agricultural employment occupies a prominent position in the local economy. However, we know that application in other contexts requires considering local specificities, such as policies and level of agricultural mechanization. For future research, we suggest investigating specific impacts of agricultural crops and including technology and education variables, which influence the resilience of the sector in the face of crises.

**Suggestion 10:** “ *We all know that the epidemic began in 2019. The data you are using starts from 2018. In my opinion , the starting year of this data should be earlier than 2018, and the ending year should be later than 2021 ( compared to August 2024 when I received this manuscript ).*

**Answer:** We chose a time frame starting in 2018 basically to avoid distortions in the results resulting from the significant economic crisis that Brazil went through between 2014 and 2016. In terms of Gross Domestic Product, the contraction of the Brazilian economy in 2015 ( [estimated at 3.62%](https://g1.globo.com/economia/mercados/noticia/2015/12/mercado-aumenta-previsao-de-queda-do-pib-para-362-em-2015.html) ) was the [worst in 25 years](https://g1.globo.com/economia/noticia/2015/11/governo-passa-prever-contracao-de-31-para-o-pib-deste-ano.html) . According to the [IBGE](https://biblioteca.ibge.gov.br/visualizacao/periodicos/1719/pia_2018_v37_n1_empresa_informativo.pdf) , between 2013 and 2016, the crisis led to the closure of 13.8 thousand industries in the country.

Regarding the period after 2021, there was no data availability at the time of analysis.

**Suggestion 11:** “ *Please provide an explanation of the sample size .”*

**Answer: We have added a description of the sample to the “ Data description ”** section .

“The research sample is made up of all municipalities in Brazil (5,570), organized in a balanced panel covering 4 years (2018-2019), totaling 22,280 observations.”

**Suggestion 12:** “ *Figure 1 in the manuscript clearly illustrates the relevant content . I suggest adding a graph to illustrate the changes ( often called △ /delta) rather than absolute values .* ”

**Answer:** We accepted the suggestion and inserted a third map in the figure, highlighting the variation in agricultural employment in the territory during the period.

**Suggestion 13:** “ *In Table 5, you have mentioned the “x2”. Do you mean “χ2”? It is called “Chi- square ”.* ”.

**Answer:** In Table 5, the term “x2” has been corrected to “χ2”, representing the “ Chi- square” test.

**Response to Reviewer 3's Comments:**

**Suggestion 1:** “ Introduction : *The research question needs to be further clarified . For example , what is the extent of the impact of the COVID-19 pandemic on agricultural in Brazil （ Not just employment rates, because you want to highlight the value of your research ） ? Which factors have the most significant impact on agricultural employment ?”*

**Answer:** In order to meet this suggestion, we have reformulated the introduction. We would like to highlight that the study examines not only employment rates, but also the impact of several factors on the Brazilian agricultural sector during the pandemic. Initially, we highlight the relevance of the agricultural sector in Brazil, including economic and employment data. In addition, we make it clear that the determinants of agricultural employment, such as public policies, climate conditions, and responses to COVID-19, are analyzed in greater depth in the theoretical framework.

**Suggestion 2:** “ Literature Review:

*It is recommended to supplement some international comparative studies on the impact of the COVID-19 pandemic on agricultural employment and research on the application of spatial econometric models in agriculture employment studies . Compare the differences between their studies and the advantages of ours .*

*Consider categorizing the literature review by research themes , such as the impact of the COVID-19 pandemic on agricultural employment , factors influencing agricultural employment , and the application of spatial econometric models.”*

**Answer:** We organized the theoretical framework according to the suggestion. The first section deals with the impacts of the pandemic on agricultural employment, the second section addresses the application of spatial econometric models to measure this impact, the third section addresses the determinants of agricultural employment, and the last section presents the characteristics of agriculture by region in Brazil. This last section was included to support the heterogeneity analysis adopted.

**Suggestion 3:** “ *Research Methodology : It is recommended to detail the construction method and selection rationale for the spatial weight matrix , as well as the specific steps and results of model selection .”*

**Answer:** We have added two justifications for choosing the spatial matrix in the specific section. Regarding the choice of the spatial model, we have completely reformulated the section to provide greater clarity in the criteria used.

**Suggestion 4:** “ *4. Research Data:*

*The research data is comprehensive but still needs further improvement . It is recommended to explain the data collection method , the reliability of the data source , and the handling method of missing data.*

*Consider adding some methods for evaluating data quality , such as descriptive statistical analysis and data cleaning ”*

**Answer:** We have accepted the suggestion **.** The "Data Selection " section has been improved to include a detailed description of the sources and collection methods, justifications for the period analyzed (2018-2021), and the treatment of missing data, using the median imputation technique to preserve the integrity of the econometric models.

**Suggestion** 5 : “ *Model Selection:
It is recommended to explain the reasons for choosing the Spatial Durbin Model and the robustness test of the model selection results.
Consider adding an introduction to other model selection methods , such as cross-validation and information criteria .”*

**Answer:** The suggestion has been addressed, so the "Model Selection " section has been improved with detailed explanations on choosing the *Spatial model. Durbin Model (SDM* ), as well as the inclusion of robustness tests, such as the Durbin test. *Hausman* , and diagnostic methods for spatial dependence.

**Suggestion 6:** “ *Research Results :*

*It is recommended to explain the economic interpretation of the research results and the implications for theory and practice .*

*Consider adding some robustness tests of the research results , such as sensitivity analysis and heterogeneity analysis .”*

**Response:** By considering these suggestions, we sought to improve the Results and Discussion and “Heterogeneity and Robustness Tests” sections . We included an economic interpretation of the results, showing the impact of COVID-19 and income transfers on agricultural employment, highlighting implications for public policies. We also added robustness tests, such as regional heterogeneity and sensitivity analysis, confirming the consistency of the results found and highlighting the occurrence of significant regional variations .

**Suggestion 7.** *” Heterogeneity Analysis :
It is recommended to add heterogeneity analysis , such as group analysis by region , agricultural type , and labor type , to reveal whether the impact of the COVID-19 pandemic on agricultural employment in Brazil varies* .”

**Answer:** We have included the “ Heterogeneity and Robustness Tests” section. In it, contain In addition to the heterogeneity test , the robustness test with a new dependent variable - the ratio between agricultural wage mass and total wages paid - to confirm the consistency of the observed impacts.

**Suggestion 8** – *“ Conclusion :
The conclusion is concise but still needs further improvement . It is recommended to summarize the main findings of the research and point out the future research directions .”*

**Response:** The suggestion was taken into account, and the conclusion section was completely reworked to highlight the main findings and present them in a clearer and more objective manner. In addition, we expanded the suggestions for future research, highlighting areas such as detailed analysis by crop type and the influence of emerging technologies in the agricultural sector.

“ ***Additional Suggestions :***

*It is recommended that the authors carefully review the paper format to ensure compliance with the journal requirements .*

*It is recommended that the authors strengthen the English expression of the paper to improve readability . This paper has research value but still needs improvement .*

*It is hoped that the authors can review the paper according to the comments of the reviewers to improve the quality of the paper* .”

**Response:** In addition to the other suggestions, we have also implemented the additional recommendations. We have carefully reviewed the entire paper, which we believe has added value to the study and brought the format and clarity of the text into line with the journal's requirements.
