# Supplementary material for: Determinants of agricultural employment during the COVID-19 pandemic: A spatial analysis of Brazilian municipalities
Source: PLoS One. 2025 Jan 9;20(1):e0316489. doi: 10.1371/journal.pone.0316489 (PMC11717285; doi:10.1371/journal.pone.0316489)
Supplement: Attachment — Submitted filename: Reviewer Comments.docx [file pone.0316489.s003.docx]

**Reviewer Comments**

This paper investigates the impact of the COVID-19 pandemic on agricultural employment in Brazil, employing spatial econometric models to analyze the relevant factors. The research holds both theoretical and practical significance, but there are several areas for improvement.

**Specific Comments**

1. **Introduction**:
   - The research question needs to be further clarified. For example, what is the extent of the impact of the COVID-19 pandemic on agricultural in Brazil（Not just employment rates, because you want to highlight the value of your research）? Which factors have the most significant impact on agricultural employment?
   - Consider adding an introduction to the current status of agricultural development in Brazil to better understand the research background.
2. **Literature Review**:
   - It is recommended to supplement some international comparative studies on the impact of the COVID-19 pandemic on agricultural employment and research on the application of spatial econometric models in agricultural employment studies. Compare the differences between their studies and the advantages of ours.
   - Consider categorizing the literature review by research themes, such as the impact of the COVID-19 pandemic on agricultural employment, factors influencing agricultural employment, and the application of spatial econometric models.
3. **Research Methodology**:
   - It is recommended to detail the construction method and selection rationale for the spatial weight matrix, as well as the specific steps and results of model selection.
4. **Research Data**:
   - The research data is comprehensive but still needs further improvement. It is recommended to explain the data collection method, the reliability of the data source, and the handling method of missing data.
   - Consider adding some methods for evaluating data quality, such as descriptive statistical analysis and data cleaning.
5. **Model Selection**:
   - It is recommended to explain the reasons for choosing the Spatial Durbin Model and the robustness test of the model selection results.
   - Consider adding an introduction to other model selection methods, such as cross-validation and information criteria.
6. **Research Results**:
   - It is recommended to explain the economic interpretation of the research results and the implications for theory and practice.
   - Consider adding some robustness tests of the research results, such as sensitivity analysis and heterogeneity analysis.
7. **Heterogeneity Analysis**:
   - **It is recommended to add heterogeneity analysis, such as group analysis by region, agricultural type, and labor type, to reveal whether the impact of the COVID-19 pandemic on agricultural employment in Brazil varies.**
8. **Conclusion**:
   - The conclusion is concise but still needs further improvement. It is recommended to summarize the main findings of the research and point out the future research directions.

**Additional Suggestions**

- It is recommended that the authors carefully revise the paper format to ensure compliance with the journal requirements.
- It is recommended that the authors strengthen the English expression of the paper to improve readability.

**This paper has research value but still needs improvement. It is hoped that the authors can revise the paper according to the comments of the reviewers to improve the quality of the paper**.
